# Ensemble learning based compressive strength prediction of concrete structures through real-time non-destructive testing

**DOI:** 10.1038/s41598-024-52046-y

**Published:** 2024-01-21

**Authors:** Harish Chandra Arora, Bharat Bhushan, Aman Kumar, Prashant Kumar, Marijana Hadzima-Nyarko, Dorin Radu, Christiana Emilia Cazacu, Nishant Raj Kapoor

**Affiliations:** 1 AcSIR-Academy of Scientific and Innovative Research, Ghaziabad, 201002 India; 2 Structural Engineering Department, CSIR-Central Building Research Institute, Roorkee, 247667 India; 3 Faculty of Civil Engineering and Architecture Osijek, J. J. Strossmayer University of Osijek, Vladimira Preloga, Osijek, Croatia; 4 Faculty of Civil Engineering, Transilvania University of Brașov, 500152 Brașov, Romania

**Keywords:** Civil engineering, Mathematics and computing

## Abstract

This study conducts an extensive comparative analysis of computational intelligence approaches aimed at predicting the compressive strength (CS) of concrete, utilizing two non-destructive testing (NDT) methods: the rebound hammer (RH) and the ultrasonic pulse velocity (UPV) test. In the ensemble learning approach, the six most popular algorithms (Adaboost, CatBoost, gradient boosting tree (GBT), random forest (RF), stacking, and extreme gradient boosting (XGB)) have been used to develop the prediction models of CS of concrete based on NDT. The ML models have been developed using a total of 721 samples, of which 111 were cast in the laboratory, 134 were obtained from in-situ testing, and the other samples were gathered from the literature. Among the three categories of analytical models—RH models, UPV models, and combined RH and UPV models; seven, ten, and thirteen models have been used respectively. AdaBoost, CatBoost, GBT, RF, Stacking, and XGB models have been used to improve the accuracy and dependability of the analytical models. The RH-M5, UPV-M6, and C-M6 (combined UPV and RH model) models were found with highest performance level amongst all the analytical models. The MAPE value of XGB was observed to be 84.37%, 83.24%, 77.33%, 59.46%, and 81.08% lower than AdaBoost, CatBoost, GBT, RF, and stacking, respectively. The performance of XGB model has been found best than other soft computing techniques and existing traditional predictive models.

## Introduction

Concrete is an indispensable construction material and popular due its durability, cost-effectiveness, fire resistance, and expedited construction properties. Concrete is made by combining several components, such as cement, water, fine particles, and coarse aggregates, in precise ratios that produce the desired compressive strength (CS). Many studies have focused on estimating the properties of hardened concrete. CS is one of the fundamental qualities of concrete that designers are interested in, and relevant information can be gathered through laboratory testing. Evaluating the CS of concrete can estimate the residual capacity of the structure^1^.

Concrete specimens (cubes and cylinders) must be loaded to failure in order to directly determine the CS of concrete. Therefore, samples must be analysed in laboratories to determine the CS of the concrete. However, this is time-consuming and very costly process. To determine the in-situ CS of structural members, concrete cores can be extracted and this method is called destructive testing. This destructive method is also costly and needs the drilling of concrete cores, transportation, and dressing of concrete cores as per the codal requirement to determine the CS of concrete. To overcome this issue, in 1948 Swiss civil engineer and bridge builder Ernst O. Schmidt invented a rebound hammer (RH) to determine the CS of concrete without damaging the structural concrete^2^. Determining the properties of concrete without damaging the concrete is called non-destructive testing (NDT) or non-destructive evaluation (NDE). A brief description of NDT is available in Section "Non-destructive testing". The commonly used NDT techniques to evaluate the CS of concrete are RH and ultrasonic pulse velocity (UPV)^3^. UPV is an NDT method used to evaluate the elastic properties and integrity of materials, particularly concrete and rock. It involves the amount of the time it takes for an ultrasonic pulse to travel through a material and be reflected back by an internal defect or the opposite surface. The velocity of the pulse can then be calculated and used to determine the quality of the material. The UPV technique serves as a prevalent method for detecting damages or assessing deterioration in civil engineering infrastructures.

Reliable and accurate models are needed to evaluate the CS of concrete, it reduces time as well as cost. Various mathematical models are accessible in the previous studies to calculate the CS of concrete with RH and UPV values. The accuracy of mathematical models is very less due to the complexity of the concrete and models are based on a limited dataset. Machine learning (ML) algorithms are good to estimate the CS of concrete and are used by numerous researchers in the field of concrete technology. There are very few studies in the literature that used ML models (step-by-step regression (SBSR), an adaptive neuro-fuzzy inference system (ANFIS), gene expression programming (GEP), high correlated variables creator machine (HCVCM)-SBSR, HCVCM-GEP, and HCVCM-ANFIS, artificial neural networks (ANN), support vector machines (SVM), Linear regression (LR), lazy-learning algorithms (LLA), and tree-based learning algorithms (TBL)) to predict CS using RH and UPV tests. Some of the studies are explained below and also described in Table 1.

**Table 1:** Summary of previously established ML models to determine the CS using RH and UPV values.

S. No	References	Input parameters	Dataset	ML methods	Range of CS (MPa)	Performance of ML models
1	Shishegaran et al.^4^	RH, UPV	516	SBSR, GEP, HCVCM-ANFIS	11.11 to 55.82	R^2^ = 0.8619 (HCVCM-ANFIS)
2	Asteris and Mokos^5^	RH, UPV	209	ANN	12.16 to 52.17	R^2^ = 0.9783
3	Shih et al.^6^	RH, UPV	95	SVM	–	MAPE = 6.77%
4	Erdal et al.^7^	RH, UPV	100	LR, LLA, TBL	–	R^2^ = 0.9065 (TBL)
5	Asteris et al.^8^	RH, UPV	629	ANN, MPMR, GPR, MARS, RVM, GP	12.16 to 63.75	R^2^ = 0.9595 (ANN)

Shishegaran et al.^4^ used SBSR, ANFIS, GEP, and other three hybrid algorithms (HCVCM-SBSR, HCVCM-GEP, and HCVCM-ANFIS) to estimate the CS using NDTs. The collected dataset contains only 516 experimental values of RH and UPV. The findings of the analyzed results show that HCVCM-ANFIS outperforms all other ML models to estimate the CS of concrete. HCVCM increases ANFIS accuracy by 5%, 10%, 3%, 20% and 7% in coefficient of determination, RMSE, NMSE, MAPE, and maximum negative error, respectively. Asteris and Mokos^5^ predicted the CS of concrete obtained from RH and UPV testing using ANN. There were only 209 experimental datasets of RH and UPV values. A single and double hidden layer was used to train the ANN model. The single hidden layer with twenty-five neurons performs well when compared to the double hidden layer. The R-value of the selected ANN was 0.9891 with a RMSE value of 1.4678 MPa. Shih et al.^6^ estimated the CS of concrete with NDT by utilizing SVM. To develop and validate the SVM model, information was gathered from 95-cylinder concrete samples. In comparison to statistical regression methods, the SVM model has a greater level of precision. LR, LLA, and TBL were utilized by Erdal et al.^7^ to estimate the concrete CS using NDT results. The performance of the TBL algorithm was greater than LR and LLA.

The use of ML algorithms in the NDT of concrete structures can improve the accuracy and efficiency of the testing process. ML algorithms can be used to analyse the data collected by NDT methods such as ultrasonic testing, infrared thermography, and ground penetrating radar, to identify and classify defects in the concrete. This can help to reduce the need for costly and time-consuming manual inspections, and can also improve the ability to detect defects that might be missed by traditional NDT methods.

The distinction of the developed ML models arises from their reliance on the most comprehensive dataset among comparable studies centered on predicting CS through NDT data, as emphasized in Table 1.

Real-time NDT (RTNDT) is a promising alternative that allows for the measurement of material properties without causing any damage. RTNDT techniques use various sensors to collect data about the material's response to external loads or other stimuli and can provide information about the material's mechanical properties in real time. This study aims to evolve a model for estimating CS based on RTNDT data. The main contribution of this study is the use of an ensemble learning (EL) approach, which is a ML technique that combines the predictions of multiple models to produce a more accurate and robust prediction.

By developing an EL approach for CS prediction based on RTNDT data, this study will provide a novel solution for NDE of materials and structures that is faster, cheaper, and safer than traditional destructive testing. The results of this study will be useful for engineers, researchers, and practitioners who are interested in the development of RTNDT techniques for material evaluation. The primary contribution of this research includes: (i) to develop accurate prediction models for structural health monitoring of concrete structures, and (ii) to identify the impact of input parameters on the CS of concrete. Notably, the developed ML models exhibit performance metrics that outperform existing models, signifying superior predictive accuracy and efficacy in estimating CS from NDT data.

## Non-destructive testing

NDT refers to a range of analysis techniques used in engineering and/or science to assess the properties, integrity, and characteristics of materials, components, or structures without causing any damage or alteration to their physical properties. NDT methods are used to examine and evaluate flaws, defects, or anomalies in a non-invasive manner, ensuring the reliability, safety, and quality of the examined materials or structures^9^. The commonly used NDT testing to estimate the CS of concrete is described below:

### Rebound hammer

**Figure 1:**
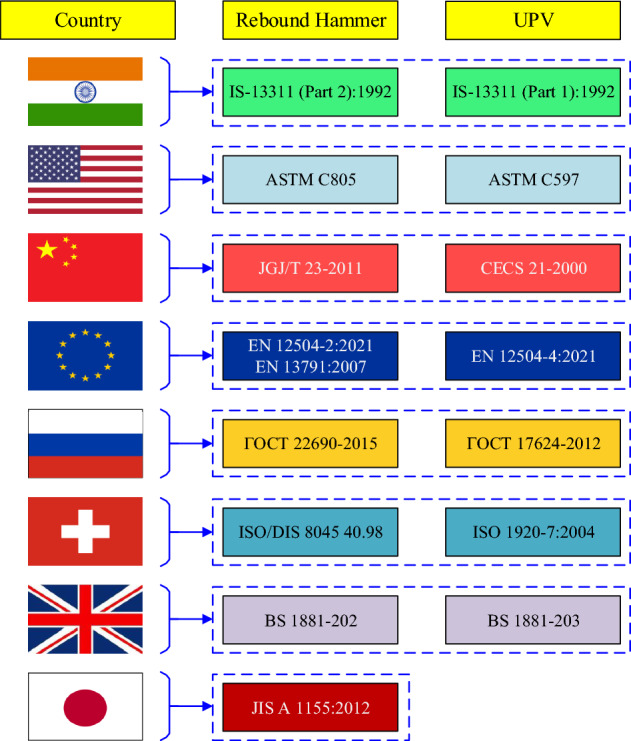
Standards of RH and UPV testing.

The RH test is one of the most widely used NDT technique for determining the CS of concrete which offers a practical and reasonably priced method to determine the concrete CS. The RH test standards are provided by various nations like India, USA, China, UK, Russia, European Union, Switzerland, and Japan, as shown in Fig. 1.

The concept behind the hardness test is that an elastic mass's rebound is influenced by how hard the surface is that it impacts. The strength of concrete is inversely correlated with the amount of energy it can absorb. The method of testing starts with carefully choosing and preparing the concrete surface that will be tested. Abrasive stones should be used to smooth up the test surface after the surface has been chosen. To impart a specific amount of energy, the hammer is then driven on the test surface.

Let the plunger make a perpendicular stroke to the surface. In the old RH, the inclination angle of the hammer has an impact on the results, but it is unimportant in the latest RH instruments. The rebound number should be recorded after the impact^10,11^. A minimum of ten readings must be taken in each area being analysed. Although there is no unique relationship between concrete hardness and strength. However, according to IS 13311 Part 2^12^, the rebound number is affected by factors such as cement type, aggregate type, carbonation of concrete, surface condition, concrete age, concrete moisture content, curing time, etc.

### Ultrasonic pulse velocity (UPV)

This method involves measuring the velocity of an ultrasonic wave propagating through a specimen to evaluate its strength and quality characteristics. A complicated system of stress waves is created as a result, including longitudinal (compressional), shear (transverse), and surface (Rayleigh) waves. The longitudinal waves, which move the quickest, are detected by the receiving transducer. The velocity of the ultrasonic wave can be used as a metric to grade the quality of the concrete, with higher velocities indicating better quality and homogeneity, and lower velocities indicating non-uniformity or the presence of defects such as cracks or voids.

**Figure 2:**
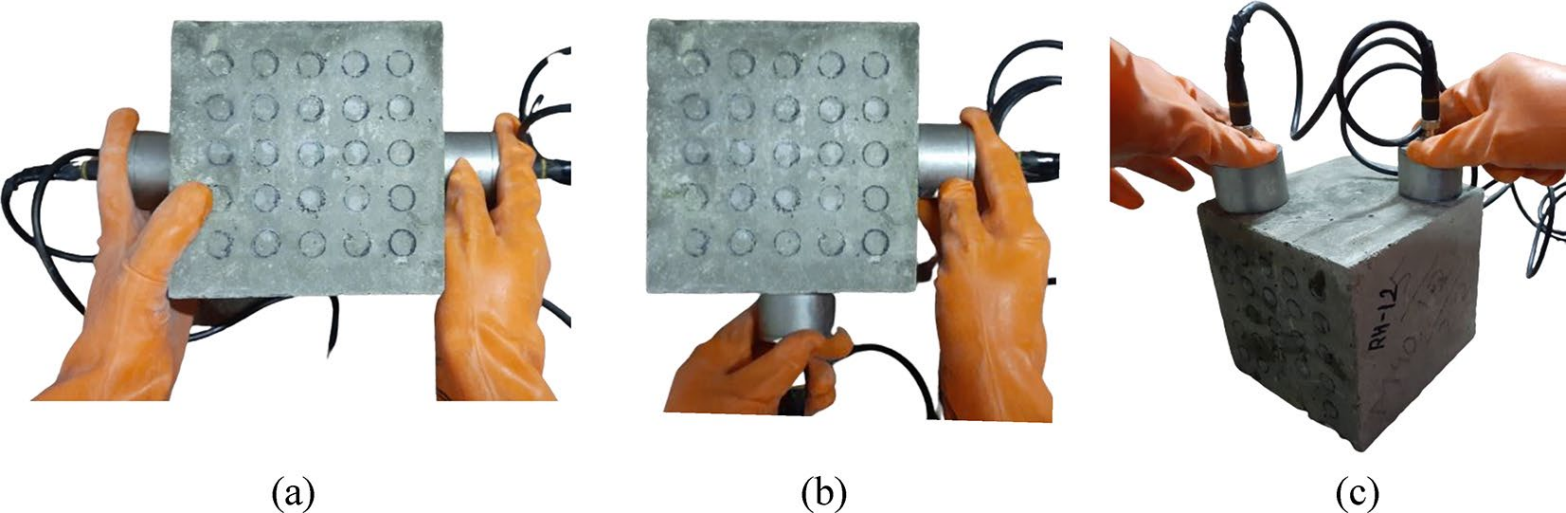
Type of UPV testing (**a**) direct test, (**b**) semi-direct test, and (**c**) indirect test.

In order to conduct this test, an ultrasonic wave pulse is introduced into the material under examination, and the elapsed time for the pulse to traverse the material is meticulously recorded. Subsequently, the pulse velocity is computed by dividing the distance, the pulse travelled within the material by the time it took for this traversal. Notably, the velocity of the ultrasonic wave is influenced by the density and elastic modulus of the material. There are various standard methods used globally to conduct the UPV test, as shown in Fig. 1. UPV testing methods can be categorized into three groups: direct testing, semi-direct testing, and indirect testing, as presented in Fig. 2.

According to IS 13311 Part 1^13^, factors that can influence the pulse velocity includes the surface conditions and moisture present in the concrete, the shape, and size of the concrete member, the temperature of the concrete, the presence of stress, the effect of reinforcing bars, etc. It is important to consider these factors to obtain accurate results. The pulse velocity (*V*) is given by:

1 $$V= \frac{L}{T}$$

where, *V, L,* and* T* are the pulse velocity, length, and effective time, respectively.

The velocity criterion for concrete quality grading according to IS 13311 Part 1^13^ is shown in Table 2, and concrete quality classification based on the RH and UPV values is shown in Table 3^14^.

**Table 2:** Velocity criterion for concrete quality grading.

Concrete quality	Excellent	Good	Medium	Doubtful
UPV value (km/s)	> 4.5	3.5 to 4.5	3.0 to 3.5	< 3.0

**Table 3:** Concrete quality classification based on RH and UPV.

RH	46	35	27	17
UPV value (km/s)	5.0	4.5	4.0	3.5
Concrete quality	Very good	Good	Fair	Poor

## Experimental methodology

### Materials

**Figure 3:**
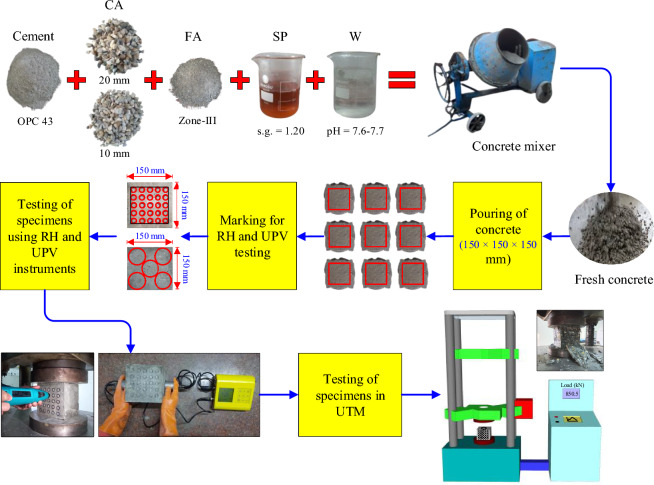
Preparation of specimens (casting, marking, NDT and compression testing).

OPC 43 grade cement was used in the present research work. The physical properties of Ordinary Portland Cement such as consistency, fineness, specific gravity, and CS after 672 ± 4 h have values of 30%, 310 m^2^/kg, 3.14, and 47.31 MPa, respectively. The coarse aggregates used in this study, measuring 20 mm and 10 mm in size, were naturally crushed and have corresponding fineness modulus of 2.25 and specific gravity of 2.71. Fine aggregates were natural with specific and fineness modulus values of 2.69 and 2.78 (Zone III), respectively. The design mix was prepared according to IS 10262: 2016^15^. Test specimens were batched onsite using different concrete mix designs with cement, water, coarse aggregate, fine aggregate, admixture, and w/c ratio, with nominal 28 days CS of 22 MPa to 44 MPa and concrete with slump flow of more than 100 mm. The total number of cast samples was 111, and each cube sample measured 150 mm × 150 mm × 150 mm. Figure 3 depicts the complete procedure of the samples from the casting phase to the testing phase.

#### CS using RH

**Figure 4:**
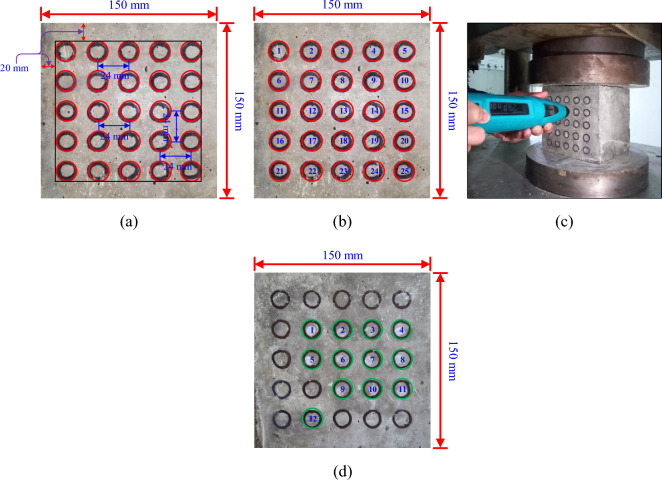
Concrete cubes (**a**) Marking of 15 mm circle for RH test (**b**) Assigning number to each circle and (**c**) Impression of RH test.

The CS of concrete can be quickly and conveniently determined using the NDT method known as the "RH test". The RH, often referred to as a Schmidt hammer, is composed of a mass that is moved along a plunger inside a tubular casing and is controlled by a spring. Before testing, all samples were taken out of the curing tank and maintained in the lab environment for roughly 24 h. Then 15 mm small circles were marked on the two faces of the concrete cube. The arrangement of the circle with the centre-to-centre spacing is presented in Fig. 4a. The total number of marking were twenty-five as shown in Fig. 4b. The concrete cube specimens were placed in a compression testing machine and subjected to a constant load of approximately 7 N/mm^2^ (based on impact energy of the hammer) (Fig. 4c). The rebound number was measured, and the CS is calculated according to IS 516:1959^16^. In the RH test, 10 to 12 rebound number responses from each test location were measured on two faces of the cube specimen (Fig. 4d).

The average rebound value of each test region is derived using an algorithmic average after the maximum set and the minimum set of results^17^ have been removed.

2 $${R}_{m}= \sum_{i=1}^{10}\frac{{RN}_{i}}{10}$$

where, ${R}_{m}$ and ${RN}_{i}$ are the average value of each test area and the measured value of each impact point, respectively.

#### Density using UPV

**Figure 5:**
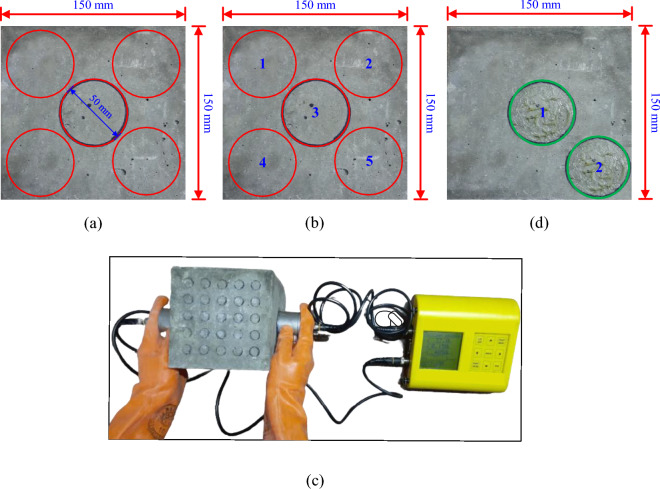
Concrete cubes (**a**) Marking of 50 mm circle for UPV test (**b**) Assigning number to each circle (**c**) testing of samples with UPV instrument, and (**d**) Impression of UPV test.

The working principle and related details of UPV are already mentioned in the subsection "Ultrasonic pulse velocity (UPV)". In the UPV test, marking has been done on two faces of the concrete cube. The diameter of the probe is 50 mm and on each face, five markings were made as shown in Fig. 5a and b. On an average only two readings were taken from the selected two faces as shown in Fig. 5d. The 54 kHz probes were used and the method of testing is a direct method or direct testing as shown in Fig. 5c.

The average UPV value can be obtained by using below expression:

3 $${UPV}_{m}= \sum_{i=1}^{2}\frac{{UPV}_{i}}{2}$$

where, ${UPV}_{m}$ and ${UPV}_{i}$ are the average value of each test area and the measured value of each point, sequentially.

#### CS using UTM

**Figure 6:**
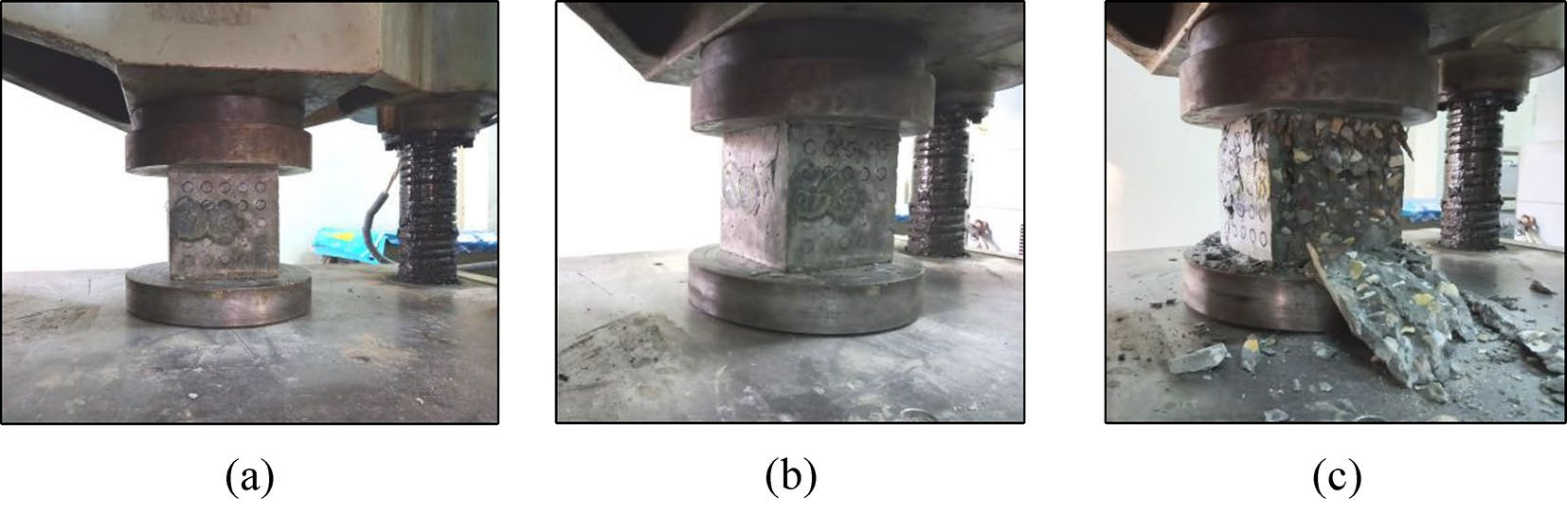
Testing of the specimen under UTM (**a**) setting of the specimen under UTM, (**b**) application of load, and (**c**) failure of the specimen.

After the RH and UPV testing, the sample had been tested under the universal testing machine (UTM) according to IS 516: 1959^16^. Load should be applied gradually and continuously without shock. Gradually increase the load until the cube either reaches its peak capacity or shows signs of cracking. The maximum load at which the cube fails should be recorded along with the type of failure (crushing, splitting, etc.) as shown in Fig. 6. IS 516 code provides guidelines for the testing of concrete cubes and the procedure is followed to obtain accurate results.

### Collected database

**Figure 7:**
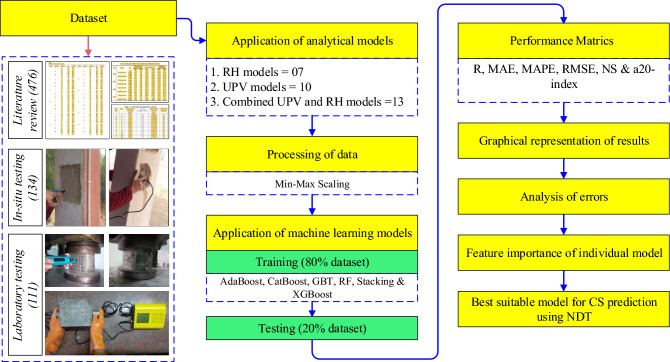
Methodology chart.

To collect the RH and UPV data of concrete cubes, a thorough research of the literature was conducted. From the published studies, 476 datasets had been gathered^5,18–21^. From in-situ NDT, 134 datasets had been obtained. Furthermore, a total of 111 concrete cubes were cast and subsequently subjected to laboratory testing employing methods such as RH, UPV, and UTM. In the end, 721 datasets were chosen to construct the ML models. Figure 7 shows the full approach used to accomplish the goal of this study. Table 4 presents the statistical characteristics (minimum, maximum, mean, standard deviation (SD) and kurtosis (K_u_)) of the gathered, in-situ, laboratory tests, and the merged dataset.

**Table 4:** Statistical parameters of all the datasets used to develop ML models.

Variable	Symbol	Units	Min	Max	Mean	SD	K_u_
From literature							
Rebound number	RN	–	14	55.5	28.68	7.91	3.70
Ultrasonic pulse velocity	UPV	km/sec	3.39	5.22	4.43	0.34	3.06
Compressive strength	CS	MPa	5.20	59.33	27.32	10.43	3.16
In-situ testing							
Rebound number	RN	–	25.30	52.80	41.36	6.08	2.72
Ultrasonic pulse velocity	UPV	km/sec	1.83	5.14	3.66	0.64	2.55
Compressive strength	CS	MPa	17.89	55.60	30.66	11.81	2.41
Laboratory testing							
Rebound number	RN	–	25.40	42.20	33.32	3.47	3.26
Ultrasonic pulse velocity	UPV	km/sec	3.21	4.56	4.14	0.15	4.71
Compressive strength	CS	MPa	22.07	44	33.48	4.70	2.67
Whole dataset							
Rebound number	RN	–	14	55.50	31.75	8.58	2.90
Ultrasonic pulse velocity	UPV	km/sec	1.8	5.22	4.24	0.49	3.97
Compressive strength	CS	MPa	5.2	59.33	30.29	9.72	2.53

**Figure 8:**
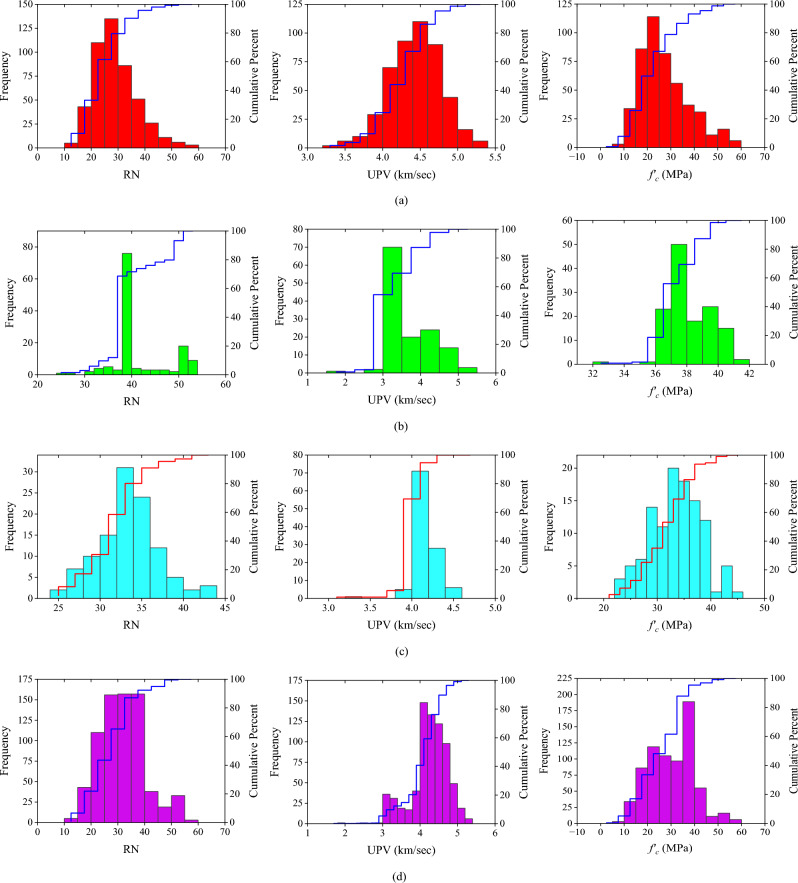
Histogram probabilities plot (**a**) data from the literature, (**b**) in-situ data, (**c**) laboratory data, and (**d**) combined all data.

A probability histogram is a graph that lists all possible outcomes along the x-axis and the likelihood of each outcome on the y-axis. The probability distribution is depicted graphically in Fig. 8.

### Processing of data

Data processing is an important step in ML algorithms. Data standardization is the process of transforming data into a common format or scale so that it can be easily compared and combined with other data. This is particularly important when working with data from different sources, as each source may have its format or scale. In this work, “Min–Max Scaling” has been used to normalize the input and output datasets. Standardization/normalization of the dataset is important because it allows for more accurate and fair comparisons between data and can improve the performance of ML algorithms^22,23^.

## Prediction models

To predict the CS using NDT methods; mathematical and ML models have been applied. The mathematical models have been divided into three categories: (a) prediction models based on RH, (b) prediction models based on UPV value, and (c) prediction models based on combined RH and UPV values. Ensemble-based ML algorithms namely AdaBoost, CatBoost, GBT, RF, stacking, and XGB have been applied to develop the CS prediction models. Detailed information on the mathematical and ML models is available in subsequent sections.

### Mathematical models

In this section, the details of analytical models based on RH, UPV, and combined RH and UPV are given in Tables 5, 6 and 7, sequentially with the year of publication. These models are the most widely acknowledged empirical connections for calculating the CS of concrete using NDT techniques that can be found in the literature. These equations rely on UPV, RH, or a combination of both for measurements, but they tend to demonstrate considerable deviation, leading to predicted results that significantly differ from the actual values.

**Table 5:** List of analytical models based on RH.

S. No	References	Year	Model name	Equation
1	Logothetis^24^	1978	RH-M1	$0.0981\left(-9.40+0.52RN+0.02{RN}^{2}\right)$
2	Trezos et al.^25^	1993	RH-M2	$0.0147{RN}^{2}+1.058RN-14.796$
3	Kheder^26^	1999	RH-M3	$0.4030{RN}^{1.2083}$
4	Qasrawi^27^	2000	RH-M4	$1.353RN-17.393$
5	Nash't et al.^28^	2005	RH-M5	$0.788{RN}^{1.03}$
6	Erdal^29^	2009	RH-M6	$-0.0177{RN}^{2}+2.0481RN-19.303$
7	Shariati et al.^30^	2011	RH-M7	$1.7206RN-26.595$

**Table 6:** List of analytical models based on UPV.

S. No	References	Year	Model name	Equation
1	Logothetis^24^	1978	UPV-M1	$0.0981\left(176.9-96.467UPV+13.906{UPV}^{2}\right)$
2	Trezos et al.^25^	1993	UPV-M2	$28.9{UPV}^{2}-221.6UPV+440.1$
3	Kheder^26^	1999	UPV-M3	$1.2\times {10}^{-5}{\left(1000UPV\right)}^{1.7447}$
4	Qasrawi^27^	2000	UPV-M4	$36.73UPV-129.077$
5	Turgut^31^	2004	UPV-M5	$1.14{e}^{0.77UPV}$
6	Nash't et al.^28^	2005	UPV-M6	$1.19{e}^{0.715UPV}$
7	Trtnik et al.^32^	2009	UPV-M7	$0.085{e}^{1.2882UPV}$
8	Erdal^29^	2009	UPV-M8	$-16.777{UPV}^{2}-167.29UPV-377.18$
9	Shariati et al.^30^	2011	UPV-M9	$15.533UPV-34.58$
10	Al-Numan et al.^33^	2015	UPV-M10	$11.804{e}^{0.2601UPV}$

**Table 7:** List of analytical models based on combined RH and UPV.

S. No	References	Year	Model name	Equation
1	Logothetis^24^	1978	C-M1	$0.0981{e}^{1.78ln\left(UPV\right)}+0.85ln\left(RN\right)-0.02$
2	Bellander^34^	1979	C-M2	$-25.568+0.000635{RN}^{3}+8.397UPV$
3	Tanigawa et al.^35^	1984	C-M3	$-0.544+0.745RN+0.951UPV$
4	Arioglu and Manzak^36^	1991	C-M4	$18.6{e}^{0.515UPV+0.019R}0.0981$
5	Kheder^26^	1999	C-M5	$0.0158{\left(1000UPV\right)}^{0.4254}{RN}^{1.1171}$
6	Nash't et al.^28^	2005	C-M6	$0.356{RN}^{0.866}{e}^{0.302UPV}$
7	Turgut and Kucuk^37^	2006	C-M7	$-194+0.77RN+44.8UPV$
8	Dolce et al.^38^	2006	C-M8	$8.925\times {10}^{-11}{\left(1000UPV\right)}^{2.6}{RN}^{1.4}$
9	Erdal^29^	2009	C-M9	$0.42RN+13.166UPV-40.255$
10	Shariati et al.^30^	2011	C-M10	$0.0981\left(-173.04+131RN+57.96UPV+{4.07UPV}^{2}\right)$
11	Huang et al.^39^	2011	C-M11	${\left(1.26+0.00015{RN}^{2}+0.035{UPV}^{3}+0.8024\right)}^{2}$
12	Nikhil et al.^40^	2015	C-M12	$1.6411\times {10}^{-9}{\left(1000UPV\right)}^{2.29366}{RN}^{1.30768}$
13	Amini et al.^41^	2016	C-M13	$\left(0.10983+0.00157RN-0.79315\left(\frac{UPV}{10}\right)-0.00002{RN}^{2}+1.29261{\left(\frac{UPV}{10}\right)}^{2}\right)\times {10}^{3}$

The CS of concrete has been adjusted using a tiny correction in the analytical models (UPV-M8, CM-10, and CM-13). In the UPV-M8, C-M10, and C-M13 models, the correction factor in the existing model is divided by 10000, 10, and 10 values, respectively.

### ML models

EL combines diverse models: bagging (bootstrap aggregating) and boosting. Bagging trains varied model instances on different data subsets, combining predictions through voting or averaging. Boosting trains sequential weak models, each correcting the previous one's errors, combining predictions with weighted emphasis. Models like random forest (RF), gradient boosting trees (GBT), AdaBoost, and XGBoost (XGB) are prominent for their superior performance and lower overfitting risks. This study applied six EL models to enhance accuracy of the existing model, showcasing EL's capability to improve predictive results. The overview of the ML models is given in Table 8.

**Table 8:** Description of ML models.

Model	Description	Year proposed	Notable features
AdaBoost	Combines weak learners iteratively by adjusting instance weights; enhances accuracy in regression and classification^42–44^	1997	Sequential training, weighted majority vote, simplicity, and effectiveness
CatBoost	Developed by Yandex in 2017, CatBoost excels in handling categorical variables efficiently within gradient boosting, requiring minimal hyperparameter tuning^45,46^	2017	Efficient handling of categorical variables, permutation-based algorithm, no need for one-hot encoding, faster training times, lower memory usage, built-in handling of missing values, automatic handling of class-imbalanced datasets
GBT	Assembles weak prediction models (like decision trees) iteratively, optimizing various loss functions; offers versatility in optimization^47,48^	1999	Adaptable to differentiable loss functions, various algorithmic variants like XGB and LightGBM, effectiveness in large-scale data, competitions, and handling large datasets
RF	Integrates multiple decision trees through EL, minimizing overfitting by training on different data subsets and features^49,50^	2001	Utilizes randomness in data subsets and feature selection, reduced overfitting, integration of bagging and boosting methods, high accuracy, and ease of use
Stacking	Introduced by David H. Wolpert in 1992, stacking combines predictions of diverse base models using a meta-model to enhance ensemble performance^51^	1992	Meta-model integration, enhancement of overall ensemble performance, applications in diverse ML tasks like NLP, computer vision, and time series forecasting
XGB	XGB, based on gradient boosting, combines multiple weak models using decision trees, with features like regularization, parallel processing, and handling of missing values, offering effectiveness in real-world problem-solving^52,53^	2016	Gradient-based iterative model creation, decision tree usage, regularization, parallel processing, handling of missing values, and categorical variables

### Model validation

There are several metrics commonly used for model validation in regression problems, such as correlation coefficient (R), root mean square error (RMSE), mean absolute percentage error (MAPE), and mean absolute error (MAE). The R-value closes to one shows the better fit of the model and lower RMSE (approaches to zero) indicates a better fit. Nash–Sutcliffe (NS) efficiency index with a value equal to one shows a good fit between the experimental and predicted values. A higher a20-index value indicates a better fit. It is always preferable to use multiple metrics to evaluate the performance of the individual model^54–63^.

4 $$R= \frac{{\sum }_{i=1}^{N}\left({r}_{i}-\overline{r }\right)\left({s}_{i}-\overline{s }\right)}{\sqrt{{\sum }_{i=1}^{N}{\left({r}_{i}-\overline{r }\right)}^{2}{\sum }_{i=1}^{N}{\left({s}_{i}-\overline{s }\right)}^{2}}}$$

5 $$MAE= \frac{1}{N}\sum_{i=1}^{N}\left|{r}_{i}-{s}_{i}\right|$$

6 $$RMSE= \sqrt{\frac{1}{N}\sum_{i=1}^{N}{\left({r}_{i}-{s}_{i}\right)}^{2}}$$

7 $$MAPE= \frac{1}{N}\sum_{i=1}^{N}\left|\frac{{r}_{i}-{s}_{i}}{{r}_{i}}\right|\times 100$$

8 $$NS=1- \frac{{\sum }_{i=1}^{N}{({r}_{i}-{s}_{i})}^{2}}{{\sum }_{i=1}^{N}{({r}_{i}-\overline{s })}^{2}}$$

9 $$a20- index=\frac{m20}{N}$$

where, *r*, *s*, $\overline{r }$, and $\overline{s }$ are the experimental values, predicted values, mean of experimental values, and mean of the predicted values, respectively. *N* represents the number of datasets, and *m20* is the number of values obtained from measured values divided by predicted value and lies in the range of 0.8 to 1.2.

## Results and discussions

### Mathematical models

**Figure 9:**
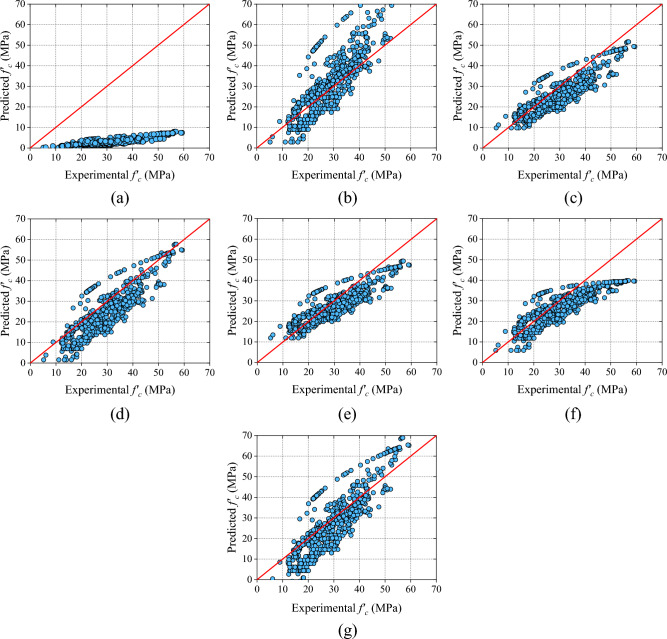
Results of analytical models based on RH (**a**) RH-M1, (**b**) RH-M2, (**c**) RH-M3, (**d**) RH-M4, (**e**) RH-M5, (**f**) RH-M6, and (**g**) RH-M7.

The mathematical models are divided into three categories namely: (i) RH models (ii) UPV models and (iii) combined RH and UPV models as mentioned in section "Prediction models". In RH models, the R-value of the RH-M2 model is the highest among all the models (RH models). But, MAE, RMSE, and MAPE values of the RH-M5 model are the lowest among all the RH models. On the other hand, NS and the a20-index of the RH-M5 hold the first position. The range of error in RH-M1, RH-M2, RH-M3, RH-M4, RH-M5, RH-M6, and RH-M7 models are  + 5.02 MPa to  + 51.94 MPa,  − 32.70 MPa to  + 13.75 MPa,  − 11.43 MPa to  + 16.96 MPa,  − 12.49 MPa to  + 16.48 MPa,  − 12.30 MPa to 16.05 MPa,  − 12.07 MPa to 19.74 MPa, and  − 17.87 MPa to 18.92 MPa, sequentially. The values of all performance metrics is shown in Table 9 and the scatter plot of all the RH models is shown in Fig. 9.

**Table 9:** Results of RH models.

Model	R	MAE (MPa)	RMSE (MPa)	MAPE (%)	NS	a20-index	SD
RH-M1	0.7962	26.0693	27.6110	90.5165	0.0298	0.0000	1.6935
RH-M2	0.7964	9.1674	12.6750	33.7092	0.7955	0.4827	18.8684
RH-M3	0.7947	5.5736	6.6975	20.0977	0.9429	0.5132	9.2628
RH-M4	0.7928	6.6096	7.8916	25.8120	0.9207	0.4313	12.4306
RH-M5	0.7931	5.1998	6.3867	19.2082	0.9481	0.6019	8.2789
RH-M6	0.7549	5.8602	7.1429	20.8040	0.9351	0.4840	8.0712
RH-M7	0.7928	7.2638	9.1434	29.3443	0.8936	0.5326	15.8079

Therefore, based on all the performance indices, it can be summarized that the precision of the RH-M5 model is good among all the RH models.

Among UPV models, the UPV-M7 model exhibits a higher R-value compared to other UPV models. However, the errors in the UPV-M7 model are higher as compared to the UPV-M6 model. The MAE value of the UPV-M6 model is 20.48% lower than the UPV-M7 model. Similarly, the RMSE and MAPE value of the UPV-M6 model is 22.71% and 15.62% lower than UPV-M7. However, the NS and a20-index of the UPV-M8 and UPV-M2 models are greater.

**Figure 10:**
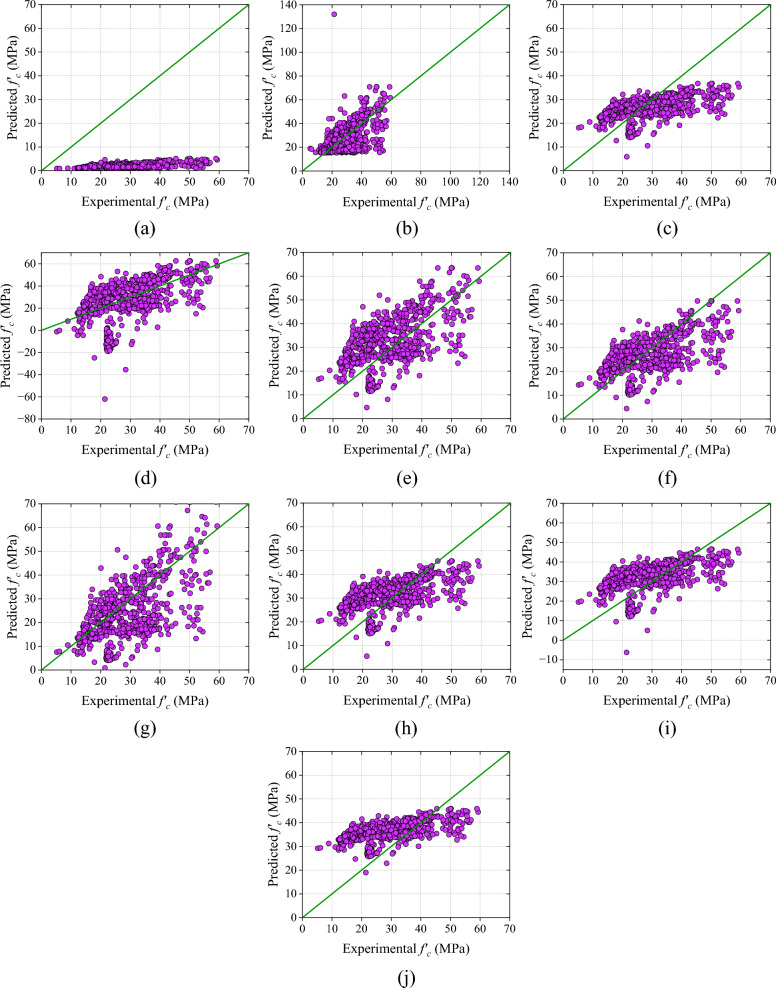
Results of analytical models based on UPV (**a**) UPV-M1, (**b**) UPV-M2, (**c**) UPV-M3, (**d**) UPV-M4, (**e**) UPV-M5, (**f**) UPV-M6, (**g**) UPV-M7, (**h**) UPV-M8, (**i**) UPV-M9, and (**j**) UPV-M10.

However, the overall performance of the UPV-M6 model is good. Therefore, it can be inferred that the UPV-M6 model outperforms other UPV models in terms of performance. A scatter plot and the values for all performance metrics are presented in Fig. 10 and Table 10 respectively.

**Table 10:** Results of UPV models.

Model	R	MAE (MPa)	RMSE (MPa)	MAPE (%)	NS	a20-index	SD
UPV-M1	0.6023	26.7906	28.5206	92.4374	− 6.0402	0.0000	0.9573
UPV-M2	0.5199	7.9779	11.1522	27.2374	− 0.0764	0.4882	12.4033
UPV-M3	0.5335	7.5141	9.2433	27.3010	0.2605	0.3800	5.3990
UPV-M4	0.5130	11.9952	15.7864	47.1140	− 1.1569	0.3273	19.589
UPV-M5	0.5823	8.8680	10.1490	36.1114	0.1085	0.3107	11.5807
UPV-M6	0.5788	7.3551	9.1297	27.1806	0.2786	0.4286	8.8651
UPV-M7	0.6058	9.2489	11.8116	32.2130	− 0.2075	0.4064	13.9663
UPV-M8	0.5398	7.5335	8.8995	31.8067	0.3145	0.3870	7.2161
UPV-M9	0.5130	8.1177	9.4867	34.8575	0.2211	0.3689	8.2843
UPV-M10	0.5412	9.4196	11.1954	43.4073	− 0.0848	0.4383	4.7237

The final category of analytical models incorporates a combination of RH and UPV measurements. The C-M6 model displays superior performance when compared to other combined models, with higher values for R-value, NS, and a20-index. Specifically, the R-value of the C-M6 model surpasses that of C-M1, C-M2, C-M3, C-M4, C-M5, C-M7, C-M8, C-M9, C-M10, C-M11, C-M12, and C-M13 by 5.99%, 6.26%, 9.30%, 7.38%, 4.01%, 29.39%, 1.59%, 13.65%, 10.94%, 52.19%, 0.97%, and 66.12%, respectively.

**Figure 11:**
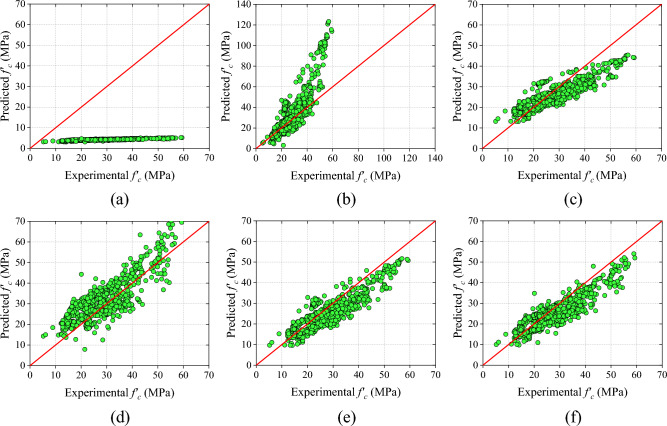

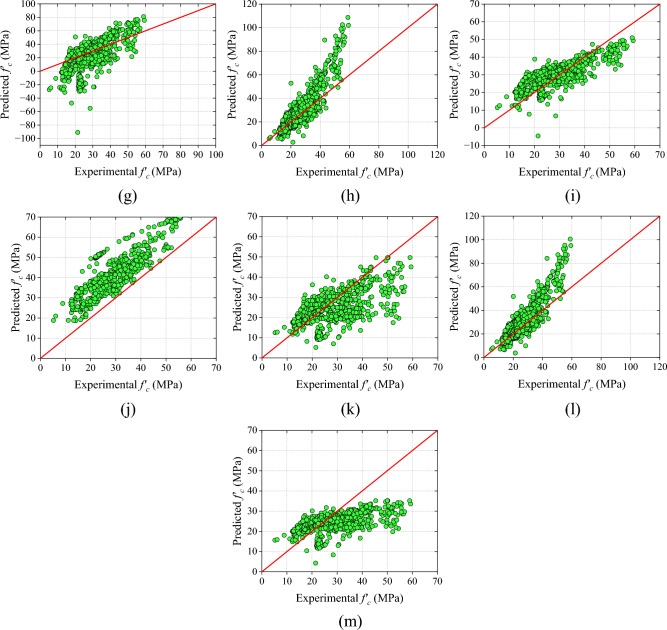
Results of analytical models based on combined RH and UPV (**a**) C-M1, (**b**) C-M2, (**c**) C-M3, (**d**) C-M4, (**e**) C-M5, and (**f**) C-M6, (**g**) C-M7, (**h**) C-M8, (**i**) C-M9, (**j**) C-M10, (**k**) C-M11, (**l**) C-M12, and (**m**) C-M13.

Overall, the C-M6 model demonstrates superior performance in comparison to combined models as well as the other two categories. Figure 11 and Table 11 depict a sequential representation of the scatter plot and performance metric values. Additionally, the C-M6 model exhibits the lowest MAE, RMSE, and MAPE values among all combined models.

**Table 11:** Results of combined RH and UPV models.

Model	R	MAE (MPa)	RMSE (MPa)	MAPE (%)	NS	a20-index	SD
C-M1	0.8460	24.7044	26.6464	83.9522	0.0090	0.0000	0.4055
C-M2	0.8439	9.7360	15.2316	31.3361	0.6762	0.4813	23.1105
C-M3	0.8204	5.3172	6.4556	18.8858	0.9418	0.5409	6.9306
C-M4	0.8351	5.4128	6.4644	21.6169	0.9417	0.6449	11.3957
C-M5	0.8621	4.9295	5.8144	17.5647	0.9528	0.5506	8.8880
C-M6	0.8967	4.3535	5.5816	14.9124	0.9565	0.6602	8.5385
C-M7	0.6930	15.3413	20.2035	63.2649	0.4303	0.2510	25.8080
C-M8	0.8827	7.8394	10.9791	25.6869	0.8318	0.4327	19.5659
C-M9	0.7890	5.2115	6.3365	20.7320	0.9440	0.6075	8.45147
C-M10	0.8083	13.3617	14.9334	54.2474	0.6887	0.1997	11.8870
C-M11	0.5892	7.5515	9.8149	26.2794	0.8655	0.4605	9.0024
C-M12	0.8880	7.1862	9.9536	23.9192	0.8617	0.5160	18.0194
C-M13	0.5398	8.0054	10.1784	26.9645	0.8554	0.3731	5.56242

### ML models

In this study, six ML models were developed and evaluated based on six distinct performance metrics. Along with these metrics, scatter plots, absolute error plots, and grouped marginal plots have also been utilized to display the accuracy and errors of the models. To compare the analytical models and the ML models, a raincloud graphic and Taylor diagram have been employed.

The AdaBoost model exhibits an R-value of 0.9280, followed by a MAPE value of 10.81%, with the NS and a20-index values being 0.8610 and 0.8724, respectively. In comparison, the CatBoost, GBT, RF, stacking, and XGB models display correlation coefficients of 0.9349, 0.9627, 0.9877, 0.9536, and 0.9970, respectively. Among all ML models, the XGB model outperforms the others in terms of R-value, with a 7.44%, 6.64%, 3.56%, 0.94%, and 4.55% higher score than AdaBoost, CatBoost, GBT, RF, and stacking, respectively. Similarly, the NS and a20-index values of the XGB model are higher than all other developed ML models. Additionally, the MAPE and MAE values of the XGB model are significantly lower than AdaBoost, CatBoost, GBT, RF, and stacking models, with reductions of 84.37%, 832.4%, 77.33%, 59.46%, and 81.08% for MAPE, and 84.99%, 83.43%, 77.60%, 60.37%, and 82.31% for MAE, respectively. Based on all performance metrics, the XGB model exhibits good precision.

**Figure 12:**
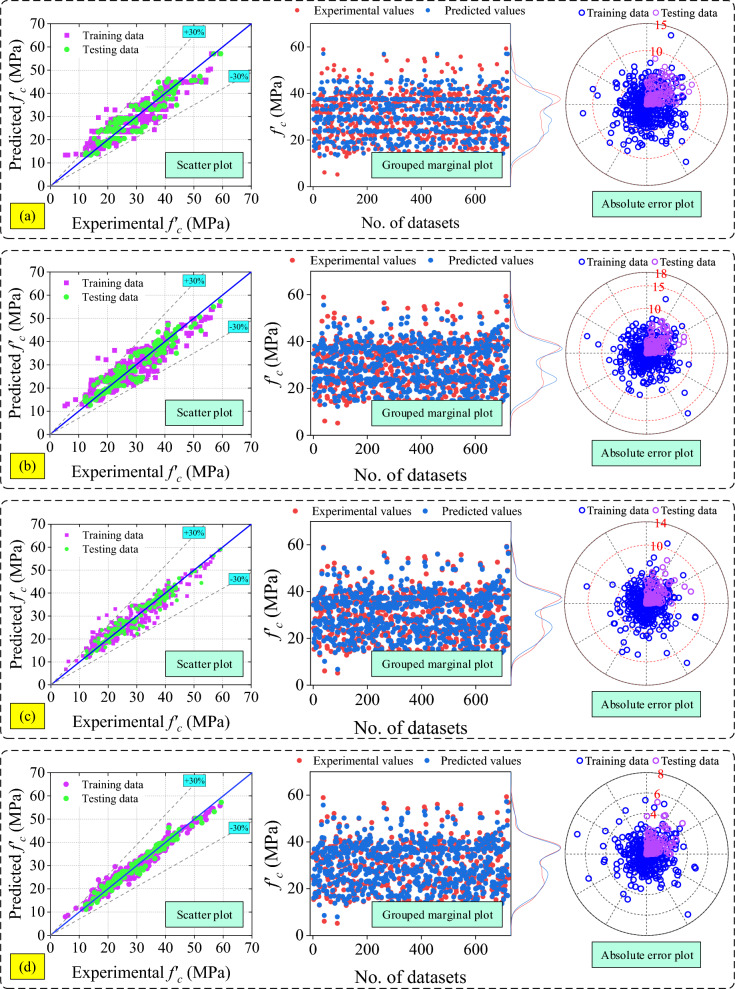

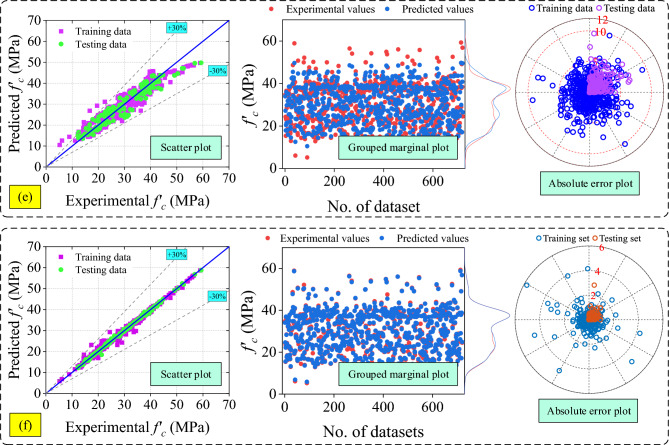
Results of the developed ML models (**a**) AdaBoost, (**b**) CatBoost, (**c**) GBT, (**d**) RF (**e**) stacking, and (**f**) XGB model.

The graphical representation of all the developed models are shown in Fig. 12a–f. Table 12 displays the values of all performance metrics for the developed models (ML models). In these figures, three plots are provided. The first plot shows the scatter plot of the training and testing dataset.

**Table 12:** Results of ML models.

Model	R	MAE (MPa)	RMSE (MPa)	MAPE (%)	NS	a20-index	SD
AdaBoost	0.9280	2.7977	3.6220	10.8137	0.8610	0.8724	8.9091
CatBoost	0.9349	2.5343	3.4498	10.0849	0.8739	0.8752	8.9411
GBT	0.9627	1.8749	2.6392	7.4540	0.9262	0.9390	9.1404
RF	0.9877	1.0595	1.5515	4.1690	0.9745	0.9847	9.2971
Stacking	0.9536	2.3739	2.9436	8.9339	0.9084	0.9501	8.9313
XGB	0.9970	0.4199	0.7576	1.6902	0.9939	0.9972	9.5838

The second plot is the grouped marginal plot, which combines a scatter plot with density curves along the margins to represent the distribution of multiple variables in a single plot. In the last plot, the absolute error values of the training and testing datasets is shown. In a grouped marginal plot, each group of observations is represented by a different colour (red colour is for experimental values and blue colour is for predicted values) and the density curves along the margins show the distribution of each variable for each group. This plot helps to visualize the relationship between two variables and the distribution of each variable for different groups in the data.

In ML models such as AdaBoost, CatBoost, GBT, RF, stacking, and XGB models only 30.37%, 33.70%, 41.61%, 61.44%, 25.38%, and 91.12% of data directly lies over the diagonal line (best-fitting line with dark blue colour) as shown in scatter plot Fig. 12a–f, sequentially. The range of the errors in the ML models are − 13.57 MPa to + 11.91 MPa, − 16.17 MPa to + 12.16 MPa, − 12.31 MPa to + 9.28 MPa, − 7.18 to 5.29 MPa, − 10.16 to 9.57 MPa, and − 5.77 MPa to + 4.79 MPa for models AdaBoost, CatBoost, GBT, RF, stacking and XGB, sequentially. The absolute error values of AdaBoost, CatBoost, GBT, RF, stacking and XGB models are 14 MPa, 17 MPa, 13 MPa, 8 MPa, 11 MPa, and 6 MPa, sequentially, as shown in Fig. 12a–f. The grouped marginal plot of the XGB model, as depicted in Fig. 12a–f, demonstrates superior performance relative to other ML models. This graphical analysis further confirms the high precision of the XGB model. Based on performance indices and graphical analysis, the ranking of all ML models is in descending order as follows: XGB, RF, GBT, stacking, CatBoost, and AdaBoost.

### Comparison between mathematical and ML models

The performance of the analytical and developed ML models had been compared with existing ML models. The metrics of all the models (existing ML models, analytical models, and developed ML model) is shown in Table 13. The performance of the XGB model is 7.39%, 0.80%, 8.09%, 25.71%, 72.25%, and 11.19% higher than Shishegaran et al.^4^, Asteris and Mokos^5^, Asteris et al.^8^, RH-M5, UPV-M6 and C-M6, sequentially. Similarly, the NS value of XGB model is 4.83%, 256.75%, and 3.91% higher than RH-M5, UPV-M6, and C-M6, sequentially. In addition to that, the a20-index of the XGB model is also higher than all the analytical as well as existing ML models. The MAPE value of the XGB model is 75.03%, 86.78%, 91.20%, 93.78%, and 88.67% lower than Shih et al.^6^, Asteris et al.^8^, RH-M5, UPV-M6 and C-M6, sequentially. In nutshell, the XGB model demonstrates superior performance as compared to both existing ML and analytical models.

**Table 13:** Comparison of existing ML models with best-fitted analytical and ML models.

Model	R	MAE (MPa)	RMSE (MPa)	MAPE (%)	NS	a20-index	SD
Shishegaran et al. (HCVCM-ANFIS)^4^	0.9284	–	35.7400	–	–	–	–
Asteris and Mokos (ANN)^5^	0.9891	–	1.4678	–	–	1	–
Shih et al. (SVM)^6^	–	–	–	6.7700	–	–	–
Asteris et al. (GPR)^8^	0.9224	4.1899	5.4815	12.7815	–	0.8204	–
RH-M5	0.7931	5.1998	6.3867	19.2082	0.9481	0.6019	8.2789
UPV-M6	0.5788	7.3551	9.1297	27.1806	0.2786	0.4286	8.8651
C-M6	0.8967	4.3535	5.5816	14.9124	0.9565	0.6602	8.5385
XGB	0.9970	0.4199	0.7576	1.6902	0.9939	0.9972	9.5838

**Figure 13:**
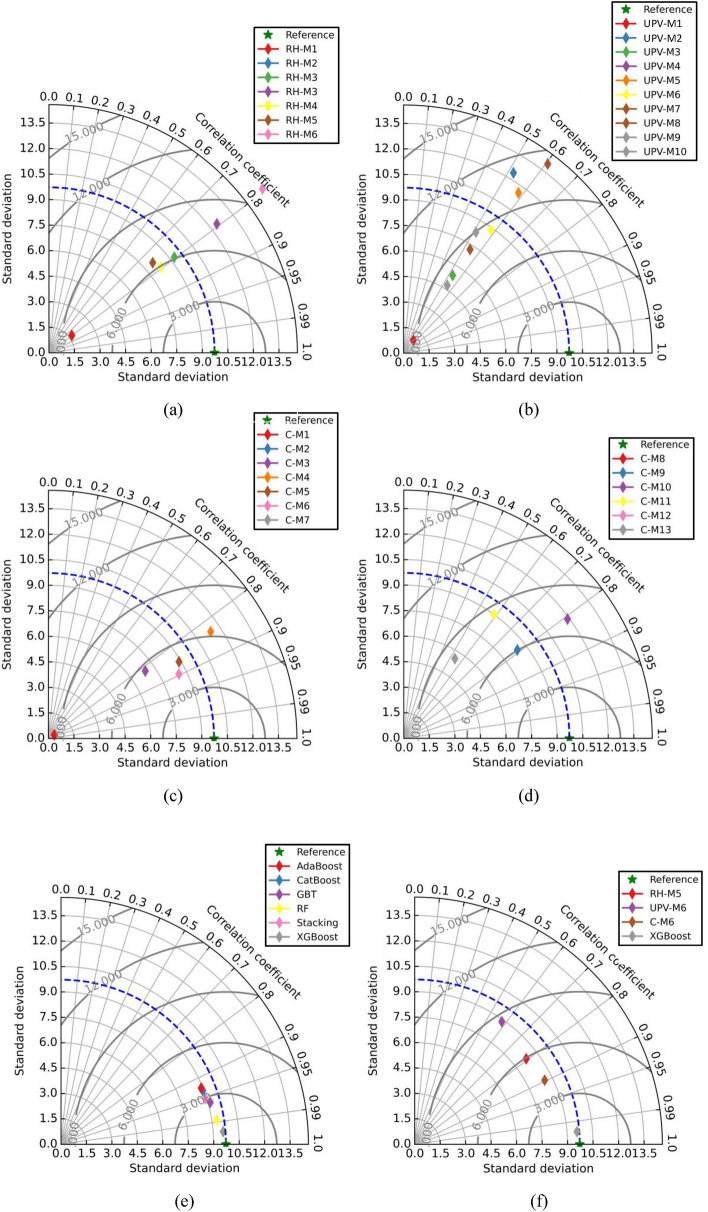
Taylor plots (**a**) RH, (**b**) UPV, (**c**) combined RH and UPV models up to C-M7, (**d**) combined RH and UPV models from C-M8 to C-M13, (**e**) developed ML models, and (**f**) best analytical (RH-M5, UPV-M6 and C-M6) and ML (XGB) models.

Taylor diagrams and raincloud plots have been used to compare how well the ML and analytical models performed. Taylor diagram is drawn between the R-value, RMSE value, and standard deviation. The dark dotted blue line represents the standard deviation of the experimental dataset and green star shows the position of the best-fitted model. Figure 13a represents the Taylor plot of RH models. In Fig. 13a, not a single model shows good fitting, because the RMSE value of all the models are above 6 MPa.

Figure 13b represents the Taylor plot of UPV models. The RMSE value of all the models above 9 MPa in Fig. 13b does not exhibit any good fitting. Figure 13c and d represent the Taylor plot of combined RH and UPV models. Only two models (C-M5 and C-M6) in Fig. 13c have RMSE values lower than six MPa. The alignment of analytical models, specifically RH-M1, RH-M2, RH-M7, UPV-M1, UPV-M4, C-M1, C-M2, C-M7, C-M8, C-M10, and C-M13, exhibits deviations from ideal placement within the Taylor plot. This deviation can be attributed to the pronounced disparity observed in the standard deviation values. The Taylor plot of the developed ML models is shown in Fig. 13e. The RMSE value of the ML models such as GBT, RF, stacking, and XGB models is less than three MPa and among these models, the XGB model shows the best fit. Figure 13f shows the Taylor plot of the best-selected analytical models (RH-M5, UPV-M6, and C-M6) and best-fitted ML model (XGB).

**Figure 14:**
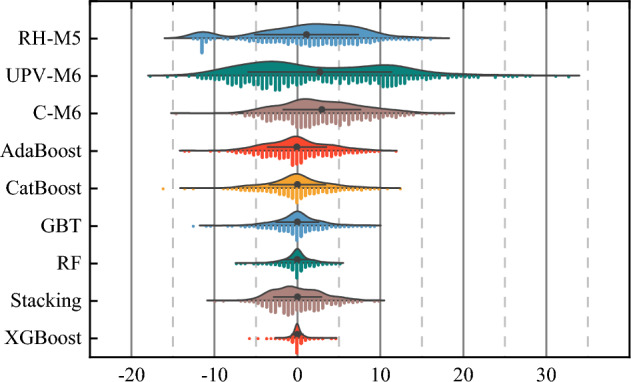
Raincloud plot to show the error comparison of best analytical models and developed ML models.

In addition to that, the raincloud plot has also been used to compare the performance of the selected analytical models (RH-M5, UPV-M6, and C-M6) and all ML models as shown in Fig. 14. The range of errors of all the models such as RH-M5, UPV-M6, C-M6, AdaBoost, CatBoost, GBT, RF, Stacking and XGB models are  − 12.30 MPa to  + 16.05 MPa,  − 17.57 MPa to  + 32.62 MPa,  − 14.75 MPa to  + 17.64 MPa,  − 13.57 MPa to  + 11.92 MPa,  − 16.17 MPa to  + 12.16 MPa,  − 12.31 MPa to  + 9.28 MPa,  − 7.18 MPa to  + 5.29 MPa,  − 10.16 MPa to  + 9.57 MPa, and  − 5.77 MPa to  + 4.79 MPa, as shown in Fig. 14. This plot also indicate that the performance of the XGB model is higher as compared to all analytical as well as developed ML models.

### Sensitivity analysis

Sensitivity analysis is a technique to determine how the uncertainty in the output of a model can be attributed to variations in its inputs. SHAP (SHapley Additive exPlanations) is a unified approach to explain the output of any ML model. It uses Shapley values, a well-established mathematical concept from cooperative game theory, to explain the output of a model by assigning a contribution to each feature^64^.

**Figure 15:**
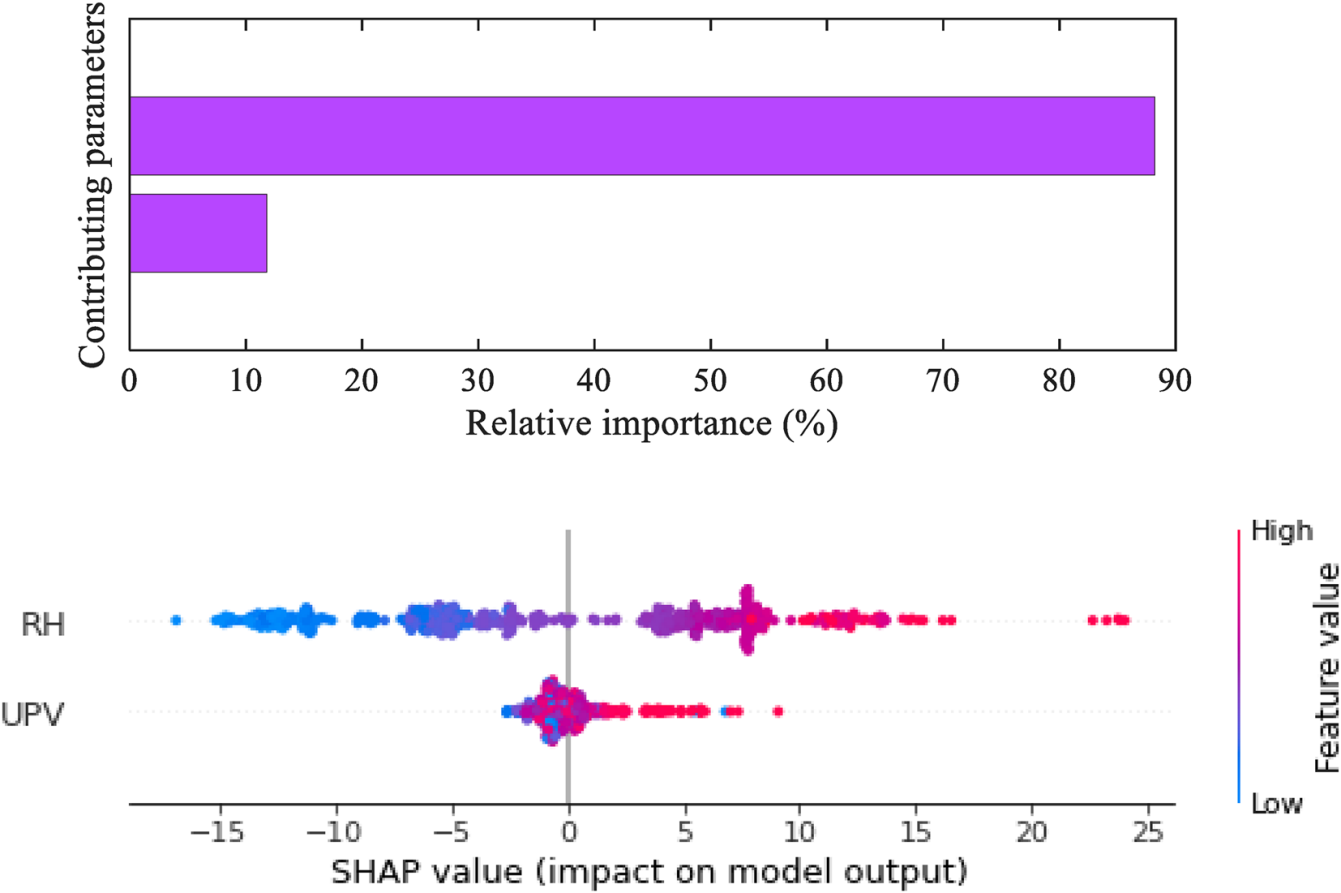
Feature importance.

For an XGB algorithm, SHAP values can be used to perform sensitivity analysis by calculating the influence of each feature to the model's predictions. Observing the magnitude and direction of the SHAP values associated with each feature enables the identification of the most influential features affecting the model's predictions. Understanding these values, helps ascertain how altering feature values will affect the predictions of the developed model. This information can be useful for interpreting the model's behaviour and for making decisions about feature selection and model interpretation. RH value has the highest impact on the CS of concrete as compared to the UPV value. The RH value has an 88.19% influence on the CS of concrete and the rest is contributed by UPV values as shown in Fig. 15.

## Conclusions

The compressive strength of concrete based on the NDT technique has been evaluated in the present study using analytical as well as the ML models. The three groups of analytical models—RH models, UPV models, and combined RH and UPV models consist of seven, ten, and thirteen models, respectively. The Ensemble-based ML algorithms (AdaBoost, CatBoost, GBT, RF, Stacking, and XGB models) have been used to enhance the accuracy of the existing models. The six performance metrics were employed to evaluate the accuracy of both analytical and ML models. Furthermore, graphical representations such as scatter plots, absolute error plots, and grouped marginal plots were utilized to analyse the fitting of the ML models. In addition to that, Taylor and raincloud plots have also been also used to compare the performance of the selected analytical and the developed ML models. Based on the performance metrics and graphical representations, the following conclusions can be drawn:

- In selected analytical models, the correlation coefficient of the RH model (RH-M5), UPV model (UPV-M6), and combined RH and UPV model (C-M6) are 0.7931, 0.5788, and 0.8967, sequentially. Similarly, the NS and a20-index of the C-M6 model are higher than RH and UPV models with values of 0.9565 and 0.6602, sequentially.
- The performance of all the developed ML models is higher than existing analytical models. Among ML models, the precision of the XGB model is higher in terms of R, RMSE, MAPE, and MAE values.
- The R-value of the XGB model is 25.71%, 72.25%, and 11.19% higher than RH-M5, UPV-ML, and C-M6 models, sequentially.
- According to the sensitivity study, RH values have a substantially larger impact on concrete's CS than UPV values.
- The Taylor and raincloud plots also confirm the reliability of the XGB model.

## Data Availability

All data generated or analysed during this study are included in this article.

## References

[1] Asteris PG, Lourenço PB, Roussis PC, Elpida Adami C, Armaghani DJ, Cavaleri L, Pilakoutas K (2022). Revealing the nature of metakaolin-based concrete materials using artificial intelligence techniques. Constr. Build. Mater..

[2] Kocáb D, Misák P, Cikrle P (2019). Characteristic curve and its use in determining the compressive strength of concrete by the rebound hammer test. Materials.

[3] Hannachi S, Guetteche MN (2012). Application of the combined method for evaluating the compressive strength of concrete on site. Open J. Civil Eng..

[4] Shishegaran A, Varaee H, Rabczuk T, Shishegaran G (2021). High correlated variables creator machine: Prediction of the compressive strength of concrete. Comput. Struct..

[5] Asteris PG, Mokos VG (2019). Concrete compressive strength using artificial neural networks. Neural Comput. Appl..

[6] Shih YF, Wang YR, Lin KL, Chen CW (2015). Improving non-destructive concrete strength tests using support vector machines. Materials.

[7] Erdal H, Erdal M, Simsek O, Erdal HI (2018). Prediction of concrete compressive strength using non-destructive test results. Comput. Concr..

[8] Asteris PG, Skentou AD, Bardhan A, Samui P, Lourenço PB (2021). Soft computing techniques for the prediction of concrete compressive strength using non-destructive tests. Constr. Build. Mater..

[9] Salin, J., Balayssac, J. P. & Garnier, V. Introduction. Non-destructive testing and evaluation of civil engineering structures. **2018**, 1–20, 10.1016/B978-1-78548-229-8.50001-7 (2018).

[10] ACI Manual of Concrete Practice. In place methods for determination of strength of concrete. Part 2: Construction practices and inspection pavements, ACI 228.1R-989, Detroit, MI, p. 25 (1994).

[11] Akashi T, Amasaki S (1984). Study of the stress waves in the plunger of a rebound hammer at the time of impact. Spec. Publ..

[12] IS 13311. Non-destructive testing of concrete–Methods of Test-Part 2: Rebound hammer.

[13] IS 13311. Non-destructive testing of concrete–Methods of Test-Part 1: Ultrasonic pulse velocity.

[14] Leslie JR, Cheesman WJ (1949). An ultrasonic method of deterioration and cracking in concrete structures. J. Proc..

[15] IS 10262. Guidelines for concrete mix design proportioning. Bur. Indian Stand. Delhi. 2009:1–21.

[16] IS 516. Indian Standard methods of tests for strength of concrete. Bur Indian Stand New Delhi, India. (1959).

[17] Xu T, Li J (2018). Assessing the spatial variability of the concrete by the rebound hammer test and compression test of drilled cores. Constr. Build. Mater..

[18] Na UJ, Park TW, Feng MQ, Chung L (2009). Neuro-fuzzy application for concrete strength prediction using combined non-destructive tests. Mag. Concr. Res..

[19] Poorarbabi A, Ghasemi M, Azhdary Moghaddam M (2020). Concrete compressive strength prediction using non-destructive tests through response surface methodology. Ain Shams Eng. J..

[20] Jain A, Kathuria A, Kumar A, Verma Y, Murari K (2013). Combined use of non-destructive tests for assessment of strength of concrete in structure. Proc. Eng..

[21] Domingo, R. & Hirose, S. Correlation between concrete strength and combined non-destructive tests for concrete using high-early strength cement. in *The Sixth Regional Symposium on Infrastructure Development* 12–13 (2009).

[22] Kumar A, Arora HC, Kapoor NR, Kumar K (2023). Prognosis of compressive strength of fly-ash-based geopolymer-modified sustainable concrete with ML algorithms. Struct. Concr..

[23] Kumar K, Saini RP (2022). Development of correlation to predict the efficiency of a hydro machine under different operating conditions. Sustain. Energy Technol. Assess..

[24] Logothetis, L. A. Combination of three non-destructive methods for the determination of the strength of concrete, PhD thesis, National Technical University of Athens, Athens, Greece, (1978).

[25] Trezos KG, Georgiou K, Marebelias C (1993). Determination of the in situ strength of concrete using the indirect methods of impact and the ultrasounds. Technika Chronika-Sci. Ed. TCG.

[26] Kheder GF (1999). A two stage procedure for assessment of in situ concrete strength using combined non-destructive testing. Mater. Struct..

[27] Qasrawi HY (2000). Concrete strength by combined nondestructive methods simply and reliably predicted. Cem. Concr. Res..

[28] Nash’t, I. H., A’bour, S. H. & Sadoon, A. A. Finding an unified relationship between crushing strength of concrete and non-destructive tests. in *Middle East Nondestructive Testing Conference & Exhibition*, Manama, Bahrain: Citeseer 27–30 (2005).

[29] Erdal M (2009). Prediction of the compressive strength of vacuum processed concretes using artificial neural network and regression techniques. Sci. Res. Essay.

[30] Shariati M, Ramli-Sulong NH, Arabnejad MM, Shafigh P, Sinaei H (2011). Assessing the strength of reinforced concrete structures through ultrasonic pulse velocity and schmidt rebound hammer tests. Sci. Res. Essays.

[31] Turgut P (2004). Evaluation of the ultrasonic pulse velocity data coming on the field. Ibis.

[32] Trtnik G, Kavčič F, Turk G (2009). Prediction of concrete strength using ultrasonic pulse velocity and artificial neural networks. Ultrasonics.

[33] Al-Numan SB, Aziz BR, Abdulla SA, Khaleel SE (2015). Compressive strength formula for concrete using ultrasonic pulse velocity. Int. J. Eng. Trends Technol..

[34] Bellander, U. NDT testing methods for estimating compressive strength in finished structures–evaluation of accuracy and testing system. in *RILEM Symp. Proc. on Quality Control of Concrete Structures*, Session **2**, 37–45 (1979).

[35] Yasuo Tanigawa, K. B. & Hiroshi, M. Estimation of concrete strength by combined nondestructive testing method. in *ACI Symposium Publication*. 82.

[36] Arioglu E, Manzak O (1991). Application of ‘sonreb’ method to concrete samples produced in yedpa construction site. Prefabr. Union.

[37] Turgut P, Kucuk OF (2006). Comparative relationships of direct, indirect, and semi-direct ultrasonic pulse velocity measurements in concrete. Russ. J. Nondestruct. Test..

[38] Dolce, M., Masi, A. & Ferrini, M. Estimation of the actual in-place concrete strength in assessing existing RC structures. in *The Second International fib Congress* 5–8 (2006).

[39] Huang Q, Gardoni P, Hurlebaus S (2011). Predicting concrete compressive strength using ultrasonic pulse velocity and rebound number. ACI Mater. J..

[40] Nikhil M, Minal BR, Deep CS, Vijay GD, Vishal TS, Shweta P (2015). The use of combined non destructive testing in the concrete strength assessment from laboratory specimens and existing buildings. Int. J. Curr. Eng. Sci. Res..

[41] Amini K, Jalalpour M, Delatte N (2016). Advancing concrete strength prediction using non-destructive testing: Development and verification of a generalizable model. Constr. Build. Mater..

[42] Freund Y, Schapire RE (1997). A decision-theoretic generalization of on-line learning and an application to boosting. J. Comput. Syst. Sci..

[43] Drucker, H. Improving regressors using boosting techniques. in *ICML '97: Proceedings of the Fourteenth International Conference on Machine Learning* **97**, 107–115 (1997).

[44] Liu J, Han X, Pan Y, Cui K, Xiao Q (2023). Physics-assisted machine learning methods for predicting the splitting tensile strength of recycled aggregate concrete. Sci. Rep..

[45] Sarkar T, Choudhury T, Bansal N, Arunachalaeshwaran VR, Khayrullin M, Shariati MA (2023). Artificial intelligence aided adulteration detection and quantification for red chilli powder. Food Anal. Methods.

[46] Hancock JT, Khoshgoftaar TM (2020). CatBoost for big data: An interdisciplinary review. J. Big Data.

[47] Sigrist F (2021). Gradient and Newton boosting for classification and regression. Expert Syst. Appl..

[48] Kulasooriya WKVJB, Ranasinghe RSS, Perera US, Thisovithan P, Ekanayake IU, Meddage DPP (2023). Modeling strength characteristics of basalt fiber reinforced concrete using multiple explainable machine learning with a graphical user interface. Sci. Rep..

[49] Breiman L (2001). Random forests. Mach. Learn..

[50] Wu F, Tang F, Lu R, Cheng M (2023). Predicting compressive strength of RCFST columns under different loading scenarios using machine learning optimization. Sci. Rep..

[51] Wolpert DH (1992). Stacked generalization. Neural Netw..

[52] Chen, T. & Guestrin, C. Xgboost: A scalable tree boosting system. in *Proceedings of the 22nd acm sigkdd International Conference on Knowledge Discovery and Data Mining* 785–794 (2016).

[53] Abdulalim Alabdullah A, Iqbal M, Zahid M, Khan K, Nasir Amin M, Jalal FE (2022). Prediction of rapid chloride penetration resistance of metakaolin based high strength concrete using light GBM and XGBoost models by incorporating SHAP analysis. Constr. Build. Mater..

[54] Suri RS, Dubey V, Kapoor NR, Kumar A, Bhushan M (2022). Optimizing the compressive strength of concrete with altered compositions using hybrid PSO-ANN. Inf. Syst. Manag. Sci..

[55] Czarnecki S, Hadzima-Nyarko M, Chajec A, Sadowski Ł (2022). Design of a machine learning model for the precise manufacturing of green cementitious composites modified with waste granite powder. Sci. Rep..

[56] Kapoor NR, Kumar A, Kumar A, Zebari DA, Kumar K, Mohammed MA (2022). Event-specific transmission forecasting of SARS-CoV-2 in a mixed-mode ventilated office room using an ANN. Int. J. Environ. Res. Public Health.

[57] Cavaleri L, Barkhordari MS, Repapis CC, Armaghani DJ, Ulrikh DV, Asteris PG (2022). Convolution-based ensemble learning algorithms to estimate the bond strength of the corroded reinforced concrete. Constr. Build. Mater..

[58] Kumar A, Arora HC, Kapoor NR, Mohammed MA, Kumar K, Majumdar A (2022). Compressive strength prediction of lightweight concrete: Machine learning models. Sustainability.

[59] Hosseinzadeh M, Mousavi SS, Hosseinzadeh A, Dehestani M (2023). An efficient machine learning approach for predicting concrete chloride resistance using a comprehensive dataset. Sci. Rep..

[60] Alakara EH, Nacar S, Sevim O, Korkmaz S, Demir I (2022). Determination of compressive strength of perlite-containing slag-based geopolymers and its prediction using artificial neural network and regression-based methods. Constr. Build. Mater..

[61] Kumar K, Saini RP (2022). Data-driven internet of things and cloud computing enabled hydropower plant monitoring system. Sustain. Comput. Inform. Syst..

[62] Nguyen N-M, Wang W-C, Cao M-T (2023). Early estimation of the long-term deflection of reinforced concrete beams using surrogate models. Constr. Build. Mater..

[63] Kumar A, Arora HC, Kumar K, Garg H (2023). Performance prognosis of FRCM-to-concrete bond strength using ANFIS-based fuzzy algorithm. Expert Syst. Appl..

[64] Kumar A, Arora HC, Kapoor NR, Kumar K, Hadzima-Nyarko M, Radu D (2023). Machine learning intelligence to assess the shear capacity of corroded reinforced concrete beams. Sci. Rep..

